# Network Pharmacology-Based Prediction of Bioactive Compounds and Potential Targets of Wenjing Decoction for Treatment of Endometriosis

**DOI:** 10.1155/2021/4521843

**Published:** 2021-06-24

**Authors:** Yu-nan Liu, Xiao-jing Hu, Bei Liu, Yu-jie Shang, Wen-ting Xu, Hui-fang Zhou

**Affiliations:** ^1^Department of Gynecology, Affiliated Hospital of Nanjing University of Chinese Medicine, Nanjing, Jiangsu, China; ^2^Nanjing University of Chinese Medicine, Nanjing, Jiangsu, China; ^3^Department of Nephrology, The First Affiliated Hospital of Henan University of CM, Zhengzhou, Henan, China

## Abstract

Endometriosis is a chronic estrogen-dependent inflammatory disorder that negatively affects the quality of life in women. The Wenjing decoction (WJD) is a traditional Chinese medicine that has been shown to have a therapeutic effect on endometriosis. Our study systematically explored the mechanism of WJD against endometriosis using a network pharmacology approach. Potentially bioactive compounds of WJD and their possible targets were retrieved from the Traditional Chinese Medicine System Pharmacology Database and Analysis Platform. The protein-protein interaction network and herbs-compounds-genes multinetwork were constructed using Cytoscape for visualization. Subsequently, the signaling pathways of common targets were retrieved from the Kyoto Encyclopedia of Genes and Genomes (KEGG) databases, and molecular docking was performed using PyRx software. In total, 48 common targets were screened, such as IL6 and ESR1, which were related to inflammation and the endocrine system. The top five bioactive compounds were quercetin, kaempferol, wogonin, beta-sitosterol, and stigmasterol. KEGG enrichment analysis revealed 65 pathways containing inflammatory- and endocrine-related signaling pathways, such as the “TNF signaling pathway” and the “estrogen signaling pathway.” Taken together, the results of our network pharmacology analysis predicted that certain active ingredients of WJD might treat endometriosis by regulating inflammation and/or endocrine, which provided references for further understanding and exploration of WJD on endometriosis.

## 1. Introduction

Endometriosis is one of the most common, benign gynecological disorders, affecting approximately 10% of women of reproductive age and 50% of those with fertility issues [1]. It is characterized by the growth of endometrial tissue (glands and stroma) outside the uterine cavity, most frequently on the ovaries, rectovaginal septum, and uterosacral ligaments [2]. Endometriosis, associated with pelvic inflammation, is most commonly manifested through dysmenorrhea, dyspareunia, gastrointestinal problems, deep pelvic pain, and infertility, which can negatively affect the quality of life among women [3, 4]. Currently, hormonal therapy and surgery are the main courses to treat endometriosis [5]. Hormonal management can inhibit tissue growth, decrease damage, and reduce pain by stopping ovulation through the use of progestins, oral contraceptives, and gonadotropin-releasing hormone agonists [6]. Laparoscopic surgery is the gold standard for the diagnosis and treatment of endometriosis, and it can be used to distinguish the disease stage and remove endometriotic tissues, such as peritoneal implants, deep nodules, and ovarian cysts. However, both management strategies can only relieve the symptoms and delay recurrence, not cure the disease fundamentally, and even with several potential side effects. Thus, the clinical benefits are controversial [7].

In China, traditional Chinese medicine (TCM) is commonly used as an alternative therapy for endometriosis due to its significant therapeutic effect and lower toxicity [8, 9]. Certain TCM formulas have been used to relieve endometriosis-associated pelvic inflammation and have achieved satisfying effects [10]. The Wenjing decoction (WJD), one of the most representative TCM prescriptions for endometriosis, has been used in Asian communities for more than 700 years. It consists of the following nine herbal materials: Radix *Angelicae Sinensis* (Danggui), Rhizoma *Ligustici* (Chuanxiong), Radix *Ginseng* (Renshen), Rhizoma *Curcumae* (Ezhu), Cortex *Moutan* (Mudanpi), Radix *Achyranthis Bidentatae* (Niuxi), Cortex *Cinnamomi* (Rougui), Radix *Paeoniae Alba* (Baishao), and Radix *Glycyrrhizae* (Gancao). The efficacy of WJD has been demonstrated in several animal and human studies, which showed that WJD could shrink existing endometriotic implants, alleviate dysmenorrhea, and increase the pregnancy rate. However, the mechanism and active ingredients of WJD have not yet been clarified [11–14].

Endometriosis is classified as an estrogen-dependent chronic inflammatory condition [15]. The local biosynthesis of estradiol by endometriotic focuses combined with inflammation in the peritoneal cavity develops an aberrant immune-endocrine microenvironment which is suitable for the growth and survival of ectopic lesions [1]. Estradiol then regulates downstream processes through nuclear estrogen receptors (ERs). Estradiol and ER signaling has been shown to be essential for lesion establishment in mouse models [16, 17]. Moreover, Burns et al. demonstrated that ERs mediate the proliferation, adhesion, and angiogenesis of ectopic lesions [18]. Inflammation also has a main role in the progression of endometriosis. During endometriotic tissue formation, inflammatory cells are recruited to the lesions, which secrete multiple inflammatory factors, including interleukin 6 (IL6), IL8, and tumor necrosis factor *α* (TNF-*α*) [19–22]. In cases of endometriosis, these inflammatory factors not only fail to effectively remove endometrial debris from menstrual blood flow in the pelvic cavity but also facilitate their implantation, hemangiogenesis, and proliferation [23]. Considering the significance of inflammation and the endocrine system in endometriosis, we hypothesize that the bioactive compounds of WJD may have a therapeutic effect in endometriosis by regulating inflammation and/or the endocrine system.

Complex TCM formulations with dozens or even hundreds of different phytochemicals can act as multitarget therapies, which affect body function by potentially employing various mechanisms that have yet to be explored. In this study, we used network pharmacology to explore the possible bioactive compounds and molecular mechanisms of WJD employed in endometriosis treatment. These results could contribute to a better understanding of the associated molecular mechanisms employed by WJD against endometriosis.

## 2. Materials and Methods

### 2.1. Identification of Target Genes Linked to Selected WJD Compounds or Endometriosis

Information about the compounds from nine herbs and their protein targets was retrieved from the Traditional Chinese Medicine System Pharmacology Database and Analysis Platform (TCMSP, http://tcmspw.com/tcmsp.php). The drug feasibility of each candidate was assessed based on oral bioavailability (OB) and drug-likeness (DL) indices assigned from the TCMSP database. A high OB value is a critical criterion for selecting biologically active molecules as candidates in drug development. DL assessments are used in drug design for determining chemical suitability and predicting pharmacodynamic and pharmacokinetic properties. In our study, molecules with OB ≥ 30% and DL ≥ 0.18 were considered active compounds with potentially beneficial pharmacological effects. After obtaining these active compounds, we used the TCMSP database and the UniProt Knowledgebase (https://sparql.uniprot.org) to obtain the corresponding target protein and gene names, respectively.

A comprehensive list of endometriosis-related genes was obtained by searching public databases, including DisGeNet (https://www.disgenet.org), Therapeutic Target Database (TTD) (http://db.idrblab.net/ttd/), and DrugBank (https://www.drugbank.ca). The overlapping genes between the bioactive compounds and the disease were identified and visualized using a Venn diagram.

### 2.2. Protein-Protein Interaction (PPI) and Multi-Network Construction and Kyoto Encyclopedia of Genes and Genomes (KEGG) Pathway Enrichment

The PPI network included information on the biological processes and molecular functions of cells. The identified overlapping target genes were introduced into the STRING network platform (https://string-db.org) with the “Homo sapiens” setting and the confidence score > 0.9 to predict the interactions. The Cytoscape software 3.7.2 (http://www.cytoscape.org/) was used to visualize the network.

The symbols of herbs and compounds in WJD, as well as the endometriosis-associated targets, were uploaded into Cytoscape software for herbs-compounds-genes multinetwork construction. In the network, different shapes of the nodes represented potential genes, active compounds, or herbs. The nodes were evaluated based on the degree, which represented the number of edges between a single node and other network nodes. The importance of a node in the network was represented by the values of this indicator, and the higher the value, the greater the importance.

The KEGG database (http://www.genome.jp/kegg/) is suitable for large-scale systematic analysis of molecular networks with interacting genes. To assess a likely mechanism of WJD in the treatment of endometriosis, the KEGG pathway enrichment analysis was performed via DAVID Bioinformatics Resources 6.8 (https://david.ncifcrf.gov). After sorting the results according to the *P* value, the top 30 significant pathway items were identified and presented via a bubble graph. Bigger and lower bubbles represented more significantly enriched pathway terms.

### 2.3. Prediction of Molecular Docking between Bioactive Compounds and Candidate Target Proteins

The bioactive composite crystal structures of the target proteins were retrieved from the Protein Data Bank (http://www.wwpdb.org), and the resulting macromolecule structure was preprocessed using PyRx 0.8 software. The molecular docking was achieved through Autodock Vina in the PyRx software. A semiempirical free energy calculation method was used to evaluate the receptor-ligand match.

### 2.4. Statistical Analysis

Fisher's exact test was used to identify significantly enriched KEGG signaling pathways. All the KEGG enrichment analysis results were sorted by *P* < 0.05 as the critical criterion, and the first 30 pathways were screened out according to the *P* value.

**Figure 1:**
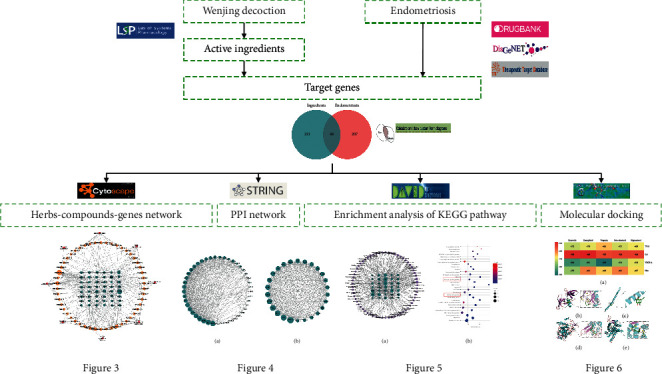
Flowchart of WJD treating endometriosis based on network pharmacology.

The flowchart of the network pharmacology approach used in this study is summarized in Figure 1.

## 3. Results

### 3.1. Bioactive Compounds and Targets of WJD

We identified 1,180 compounds of WJD in the TCMSP database, among which 171 met the screening criteria of OB ≥ 30% and DL ≥ 0.18 (the compounds of Cortex *Cinnamomi* did not satisfy the filter criteria). After removing 18 repetitive compounds, we entered the remaining compounds into the TCMSP database and identified 272 nonrepetitive target points. To obtain the gene names of these proteins, we used the UniProt Knowledgebase for the conversion, and then we identified 269 target sites. The numbers of predicted ingredients, bioactive compounds, and corresponding targets are listed in Table 1.

### 3.2. Endometriosis-Associated Target Genes and Bioactive WJD Compounds

**Figure 2:**
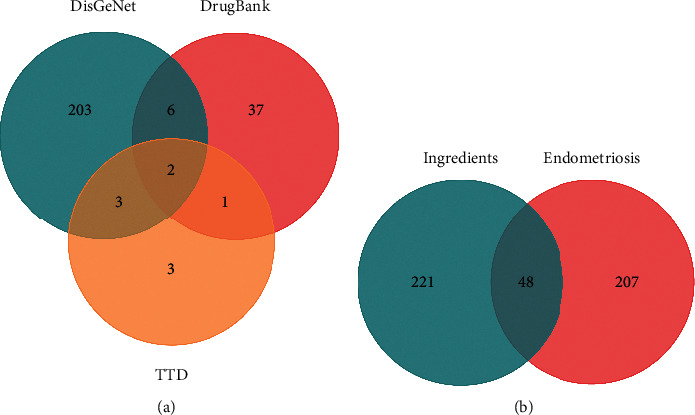
Venn diagrams. (a) Common genes from the three databases (DisGeNet, TTD, and DrugBank). (b) Overlapping target genes between WJD and endometriosis.

We retrieved 255 nonredundant endometriosis-related genes from the three databases (DisGeNet, TTD, and DrugBank) (Figure 2(a)). Mapping the endometriosis-related genes with the targets of bioactive compounds, we identified 48 intersected genes (Figure 2(b)). Detailed information about the bioactive compounds associated with the final target genes is provided in Table 2.

### 3.3. Key Bioactive Compounds of WJD against Endometriosis

**Figure 3:**
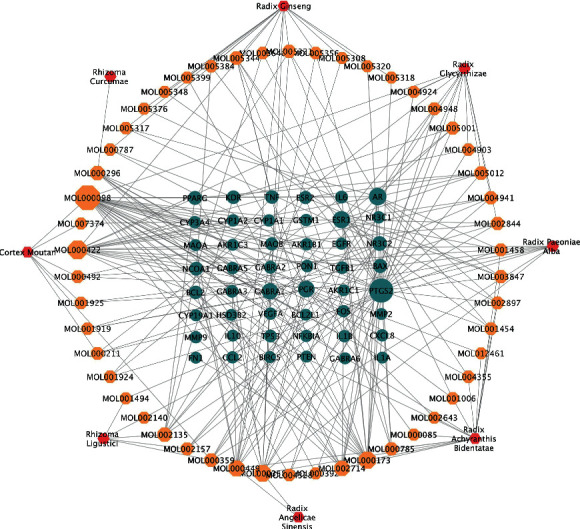
The herbs-compounds-genes multinetwork. The edges between nodes symbolize the interactions between them (more edges indicate greater relevance). Symbols: red hexagons, herbs; yellow octagons, bioactive compounds; blue circles, target genes.

The herbs-compounds-genes multinetwork included 8 herbs, 50 compounds, and 48 target genes (Figure 3). The distribution of the network connections indicated that WJD acted as a multitarget therapy against endometriosis by employing multiple bioactive compounds. Furthermore, we used the Cytoscape software to calculate the degree of the network. Seventeen compounds had a degree value exceeding the mean of all nodes (mean degree = 4.906), thereby demonstrating their pharmacological importance. The following top five active compounds were selected: quercetin (MOL000098, degree = 34), kaempferol (MOL000422, degree = 19), wogonin (MOL000173, degree = 15), beta-sitosterol (MOL000358, degree = 14), and stigmasterol (MOL00449, degree = 12).

### 3.4. Key Hub Genes in the PPI Network

**Figure 4:**
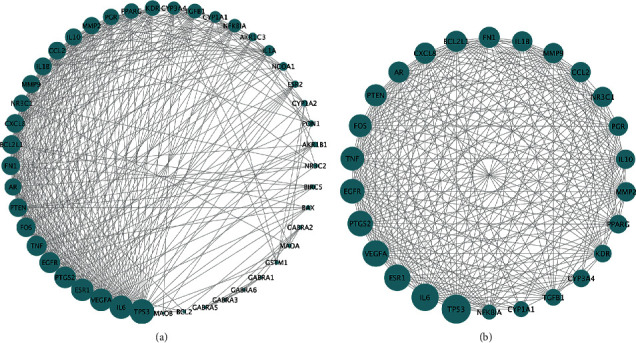
PPI network diagram of potential targets. (a) PPI network of 45 targets in the treatment of endometriosis using WJD. The blue circles mark the target proteins, and the node size corresponds to the degree value. (b) Diagram of 26 core targets.

The PPI network was obtained from the STRING database and visualized by the Cytoscape software. There were 45 nodes and 366 edges representing the target genes and the interactions between the targets, respectively (Figure 4(a)). The mean degree value of all genes estimated was 16.267, and 26 targets exceeded the average, suggesting that WJD might exert its pharmacological effects mainly through these targets (Figure 4(b)). The top four target genes based on their degree values were TP53, IL6, VEGFA, and ESR1.

### 3.5. The KEGG Pathway Enrichment Analysis and the Target-Pathway Network

**Figure 5:**
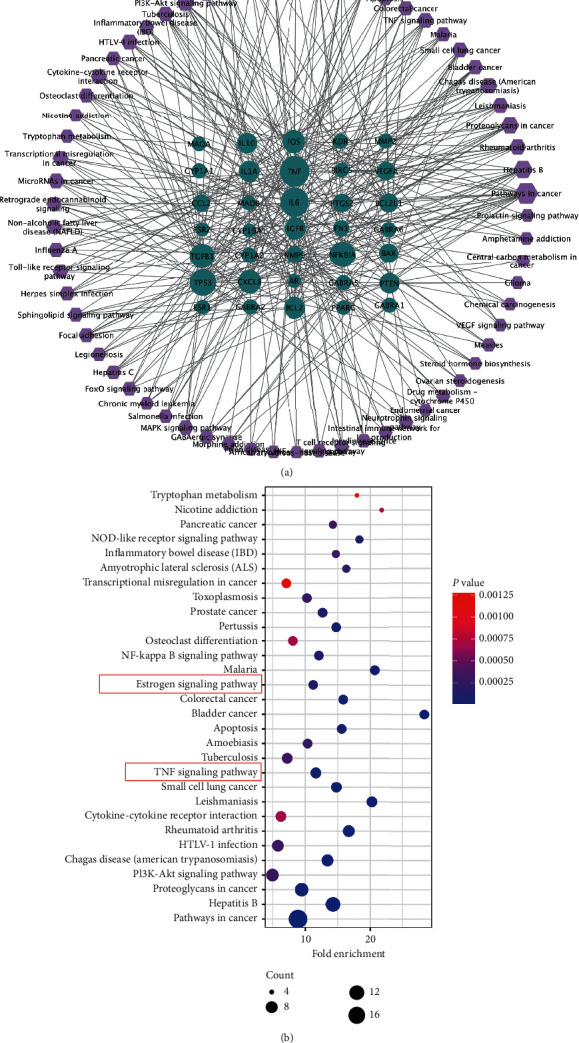
The KEGG signaling pathway diagrams. (a) The KEGG pathway enrichment analysis of 48 targets. Symbols: purple octagons, pathways; blue circles, targets. (b) Bubble diagram of the KEGG pathways. The *y*-axis shows the pathway name, and the *x*-axis indicates the percentage. The number of enriched genes in each pathway is presented via the bubble area, and the *P* value is marked by the bubble color.

Forty-eight potential targets of WJD in the treatment of endometriosis were uploaded to the DAVID database for enrichment analysis, and 65 pathways were obtained with *P* < 0.05 (Figure 5(a)). The bubble diagram shown in Figure 5(b) represents the top 30 pathways, including the inflammatory- and endocrine-related signaling pathways, such as the “TNF signaling pathway” and the “estrogen signaling pathway.”

### 3.6. Molecular Docking Predictions

**Figure 6:**
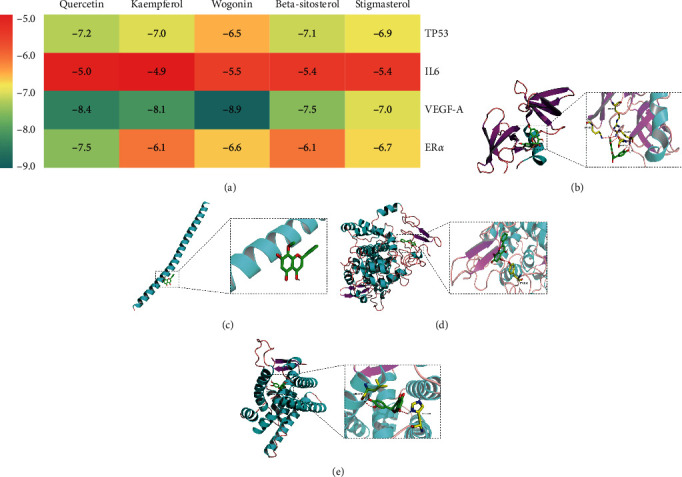
Molecular models of bioactive compounds binding to the predicted target proteins. (a) Heatmap of the binding energy by docking analysis. (b) The binding modes of TP53 and quercetin. (c) The binding modes of IL6 and wogonin. (d) The binding modes of VEGF-A and wogonin. (e) The binding modes of ER*α* and quercetin.

In general, the binding energy between ligand and receptor is less than zero, indicating that they can bind spontaneously; generally, the lower the binding energy, the stronger the combined effect affinity. We selected the top 4 target genes (TP53, IL6, VEGFA, and ESR1) that encoded the following proteins: tumor protein p53 (TP53), IL6, vascular endothelial growth factor A (VEGF-A), and estrogen receptor alpha (ER*α*). Using the selected proteins with the top 5 active compounds (quercetin, kaempferol, wogonin, beta-sitosterol, and stigmasterol), we performed molecular docking simulations. The docking results showed that these 4 targets could directly couple with the five bioactive compounds (Figure 6(a)). Furthermore, TP53 with quercetin, IL6 with wogonin, VEGF-A with wogonin, and ER*α* with quercetin displayed the strongest combined effects, as shown by the specific docking diagrams in Figures 6(b)–6(e).

## 4. Discussion

Endometriosis is a chronic, estrogen-dependent, inflammatory disease. Several theories have been proposed for its pathogenesis, including the induction theory, Mayer's coelomic metaplasia theory, and the most widely accepted Sampson's implantation theory [24]. Sampson's theory claims that the ectopic endometrial foci originate from the exfoliation of endometrial cells during menstruation and spread to the peritoneum via the retrograde tubal flow [25]. Retrograde menstruation, as a major way of dissemination of endometriotic cells, occurs in almost all women, but only a proportion of them (∼10%) develop endometriosis [7]. Hence, it appears that other factors may be involved in the development of ectopic endometriotic lesions.

Endometriosis has a distinct immune microenvironment and endocrine dysfunction, which promotes sustained proliferation, vascularization, and impaired apoptosis of the endometrial foci [26–28]. Our Venn diagram showed that the therapeutic effect of WJD on endometriosis was likely related to 48 genes (Figure 2). Furthermore, the results of the PPI network suggested that 26 targets, including IL6, ESR1, TP53, and VEGFA, were related to the occurrence and development of endometriosis, indicating that these targets might play an important role in the activity of WJD against endometriosis.

IL6, which is mainly secreted from macrophages and endometriotic cells, is involved in the inflammatory immune response. Moreover, it can inhibit apoptotic pathways, increase angiogenesis, that is, a process to develop new blood vessels and supply nutrients to growing ectopic tissues, and promote both cell adhesion and proliferation in endometriotic lesions [29]. ER*α*, an estrogen receptor, is encoded by ESR1. Not only have the levels of ER*α* been shown to be elevated in ectopic lesions, but they also promote the survival of cells in endometrial tissues by promoting cell proliferation, maintaining vascularization, and enabling cells to evade apoptosis [2]. TP53, a tumor suppressor, negatively regulates cell proliferation, inhibits angiogenesis, and induces apoptosis. In endometriotic tissues, reduction in the abundance and/or activity of TP53 frequently occurs, which promotes the uncontrolled proliferation and growth of ectopic lesions [30]. VEGF-A, a vital angiogenic factor expressed in ectopic tissues, mediates angiogenesis [31]. Based on the analysis of the top gene targets in the PPI network, we found that WJD might treat endometriosis by regulating inflammation and/or the endocrine system through IL6, ER*α*, and other cytokines.

In this network pharmacology study, we established a herbs-compounds-genes multinetwork model using the Cytoscape software and identified 50 potentially bioactive compounds of WJD, including quercetin, kaempferol, wogonin, beta-sitosterol, and stigmasterol. Quercetin, an active ingredient common to Cortex *Moutan*, Radix *Achyranthis Bidentatae*, and Radix *Glycyrrhizae*, has been previously shown to inhibit cell proliferation, induce cell apoptosis, counteract inflammation, and regulate estrogen, as well as progesterone receptors on endometriosis autoimplanted mouse models [32, 33]. Kaempferol, presented in Radix *Paeoniae Alba*, Cortex *Moutan*, Radix *Ginseng*, Radix *Achyranthis Bidentatae*, and Radix *Glycyrrhizae*, exerts anti-inflammatory effects by suppressing the release of IL6, IL1*β*, IL18, and TNF-*α* [34]. It also has an antiangiogenic effect by reducing VEGF secretion and acts as an endocrine modulator to regulate steroid receptor expression *in vivo* [35, 36]. Wogonin, an ingredient found in Radix *Achyranthis Bidentatae*, can decrease inflammatory and angiogenic hallmarks in a concentration-dependent manner [37]. Beta-sitosterol is a common compound in Radix *Angelicae Sinensis*, Radix *Paeoniae Alba*, Radix *Ginseng*, and Radix *Achyranthis Bidentatae*. In many *in vitro* and *in vivo* studies, it has been shown to possess various bioactivities, such as immunomodulatory, antimicrobial, and anti-inflammatory activities [38]. Stigmasterol, as a natural, plant-derived product with an anti-inflammatory effect, is found in Radix *Angelicae Sinensis*, Radix *Ginseng*, Radix *Achyranthis Bidentatae*, Rhizoma *Ligustici*, Cortex *Moutan*, Radix *Paeoniae Alba*, and Radix *Glycyrrhizae*. It has been reported that rats treated with stigmasterol have significantly suppressed the expression of proinflammatory mediators and increased the expression of anti-inflammatory cytokines [39]. Moreover, the molecular docking results showed that these five main compounds of WJD had a high binding affinity to the top four target proteins from the PPI network (Figure 6). Collectively, this network analysis suggested that the major active components of WJD may be effective for the treatment of endometriosis by counteracting inflammation and regulating the endocrine antiproliferation processes, along with other biological processes.

The results of the KEGG pathway analysis indicated that WJD might treat endometriosis via inflammatory- and endocrine-related signaling pathways, such as the “TNF signaling pathway” and the “estrogen signaling pathway.” Concentrations of TNF are elevated in the peritoneal fluid and serum of endometriosis patients, especially in the early stages of the disease [40, 41]. An increased TNF concentration is associated with the enhanced motility of endometrial stromal cells through the regulation of ERK1/2 signaling, and it has been shown to activate systemic and local inflammation mechanisms in the development and progression of endometriosis by increasing chemokines and proinflammatory cytokines levels [42]. On the other hand, estrogen is a necessary hormone for the proliferation and expansion of ectopic lesions [43]. In a recent study, treatment for endometriosis mainly blocks the production or function of estrogen [44]. ER*α* has a high affinity for estrogen, and its function is mainly mediated through ER*α*, which is coupled with the “estrogen signaling pathway” [45].

In this study, network pharmacology was used to illustrate that WJD might treat endometriosis by regulating inflammation and/or the endocrine system. Our results provide guidance for further investigation of the mechanism, but there are still some limitations. Firstly, the acquisition of our active compounds and disease targets is based on existing databases that may not be comprehensive. For example, the active components of cinnamon in WJD did not meet the search filter criteria of the TCSMP database. Secondly, although we have identified possible related compounds and targets of WJD against endometriosis and performed molecular docking, it is still necessary to verify the involved mechanisms *in vitro* and *in vivo*.

## 5. Conclusions

In this study, we employed a network pharmacology-based approach to identify the bioactive compounds of WJD and their potential targets in endometriosis. The results suggested that the bioactive compounds of WJD against endometriosis included 48 targeted genes, among which IL6 and ESR1 were closely related to inflammation and the endocrine system, respectively. The mechanisms employed by WJD against endometriosis were related to 65 signaling pathways, including inflammatory- and endocrine-related signaling pathways, such as the “TNF signaling pathway” and the “estrogen signaling pathway.” Thus, based on the network pharmacology analysis, we concluded that the mechanisms for treating endometriosis by WJD included the modulation of inflammation and/or the endocrine system, but *in vitro* and *in vivo* experimental validation is needed to corroborate our research further.

## Figures and Tables

**Table 1:** Basic information about WJD compounds and predicted targets.

Herbs	Number of compounds	Number of bioactive compounds	Number of predicted targets
Radix *Angelicae Sinensis*	105	2	54
Rhizoma *Ligustici*	108	7	30
Radix *Ginseng*	190	22	118
Rhizoma *Curcumae*	81	3	24
Cortex *Moutan*	55	11	173
Radix *Achyranthis Bidentatae*	176	20	187
Cortex *Cinnamomi*	100	0	0
Radix *Paeoniae Alba*	85	13	95
Radix *Glycyrrhizae*	280	93	238

**Table 2:** Information on the bioactive compounds of WJD.

Number	Molecular ID	Active ingredients	OB (%)	DL	Mapping target number	Herbs
1	MOL000358	Beta-sitosterol	36.91	0.75	10	Radix *Angelicae Sinensis*
						Radix *Paeoniae Alba*
						Radix *Ginseng*
						Radix *Achyranthis Bidentatae*
2	MOL000449	Stigmasterol	43.83	0.76	8	Radix *Angelicae Sinensis*
						Radix *Ginseng*
						Radix *Achyranthis Bidentatae*
3	MOL000359	Sitosterol	36.91	0.75	2	Rhizoma *Ligustici*
						Cortex *Moutan*
						Radix *Paeoniae Alba*
						Radix *Glycyrrhizae*
4	MOL002157	Wallichilide	42.31	0.71	3	Rhizoma *Ligustici*
5	MOL002135	Myricanone	40.6	0.51	7	Rhizoma *Ligustici*
6	MOL002140	Perlolyrine	65.95	0.27	1	Rhizoma *Ligustici*
7	MOL001494	Mandenol	42	0.19	1	Rhizoma *Ligustici*
8	MOL001924	Paeoniflorin	53.87	0.79	2	Radix *Paeoniae Alba*
9	MOL000211	Mairin	55.38	0.78	1	Radix *Paeoniae Alba*
						Cortex *Moutan*
						Radix *Glycyrrhizae*
10	MOL001919	Palbinone	43.56	0.53	2	Radix *Paeoniae Alba*
11	MOL001925	Paeoniflorin	68.18	0.4	1	Radix *Paeoniae Alba*
						Cortex *Moutan*
12	MOL000492	Cianidanol	54.83	0.24	2	Radix *Paeoniae Alba*
						Cortex *Moutan*
13	MOL000422	Kaempferol	41.88	0.24	14	Radix *Paeoniae Alba*
						Cortex *Moutan*
						Radix *Ginseng*
						Radix *Achyranthis Bidentatae*
						Radix *Glycyrrhizae*
14	MOL007374	5-[[5-(4-Methoxyphenyl)-2-furyl]methylene]barbituric acid	43.44	0.3	1	Cortex *Moutan*
15	MOL000098	Quercetin	46.43	0.28	30	Cortex *Moutan*
						Radix *Achyranthis Bidentatae*
						Radix *Glycyrrhizae*
16	MOL000296	Hederagenin	36.91	0.75	7	Rhizoma *Curcumae*
17	MOL000787	Fumarine	59.26	0.83	2	Radix *Ginseng*
18	MOL005317	Deoxyharringtonine	39.27	0.81	2	Radix *Ginseng*
19	MOL005376	Panaxadiol	33.09	0.79	1	Radix *Ginseng*
20	MOL005348	Ginsenoside-Rh4	31.11	0.78	1	Radix *Ginseng*
21	MOL005399	Alexandrin	36.91	0.75	9	Radix *Ginseng*
22	MOL005384	Suchilactone	57.52	0.56	10	Radix *Ginseng*
23	MOL005344	Ginsenoside-Rh2	36.32	0.56	5	Radix *Ginseng*
24	MOL003648	Inermin	65.83	0.54	2	Radix *Ginseng*
25	MOL005321	Frutinone A	65.9	0.34	4	Radix *Ginseng*
26	MOL005356	Girinimbin	61.22	0.31	2	Radix *Ginseng*
27	MOL005308	Aposiopolamine	66.65	0.22	1	Radix *Ginseng*
28	MOL005320	Arachidonate	45.57	0.2	1	Radix *Ginseng*
29	MOL005318	Dianthramine	40.45	0.2	1	Radix *Ginseng*
30	MOL004924	(-)-Medicocarpin	40.99	0.95	1	Radix *Glycyrrhizae*
31	MOL004948	Isoglycyrol	44.7	0.84	3	Radix *Glycyrrhizae*
32	MOL005001	Gancaonin H	50.1	0.78	4	Radix *Glycyrrhizae*
33	MOL004903	Liquiritin	65.69	0.74	2	Radix *Glycyrrhizae*
34	MOL005012	Licoagroisoflavone	57.28	0.49	5	Radix *Glycyrrhizae*
35	MOL004941	(2R)-7-Hydroxy-2-(4-hydroxyphenyl)chroman-4-one	71.12	0.18	4	Radix *Glycyrrhizae*
36	MOL002844	Pinocembrin	64.72	0.18	4	Radix *Glycyrrhizae*
37	MOL000392	Formononetin	69.67	0.21	1	Radix *Glycyrrhizae*
38	MOL004328	Naringenin	59.29	0.21	2	Radix *Glycyrrhizae*
39	MOL001458	Coptisine	30.67	0.86	3	Radix *Achyranthis Bidentatae*
40	MOL003847	Inophyllum E	38.81	0.85	4	Radix *Achyranthis Bidentatae*
41	MOL002897	Epiberberine	43.09	0.78	3	Radix *Achyranthis Bidentatae*
42	MOL001454	Berberine	36.86	0.78	3	Radix *Achyranthis Bidentatae*
43	MOL012461	28-Norolean-17-en-3-ol	35.93	0.78	1	Radix *Achyranthis Bidentatae*
44	MOL004355	Spinasterol	42.98	0.76	2	Radix *Achyranthis Bidentatae*
45	MOL001006	Chondrillasterol	42.98	0.76	2	Radix *Achyranthis Bidentatae*
46	MOL002643	Delta 7-stigmastenol	37.42	0.75	1	Radix *Achyranthis Bidentatae*
47	MOL000085	Beta-daucosterol	36.91	0.75	10	Radix *Achyranthis Bidentatae*
48	MOL000785	Palmatine	64.6	0.65	4	Radix *Achyranthis Bidentatae*
49	MOL000173	Wogonin	30.68	0.23	14	Radix *Achyranthis Bidentatae*
50	MOL002714	Baicalein	33.52	0.21	9	Radix *Achyranthis Bidentatae*

## Data Availability

The data used to support the findings of this study are available from the corresponding author upon request.
